# A preclinical randomised controlled dose optimization of megadose sodium ascorbate for reversal of gram-negative sepsis-induced cardiovascular, brain and kidney dysfunction

**DOI:** 10.1186/s13054-025-05799-5

**Published:** 2025-12-28

**Authors:** Connie Pei Chen Ow, Rachel M. Peiris, Anton Trask-Marino, Sally G. Hood, Ashenafi H. Betrie, Darius J. R. Lane, Rinaldo Bellomo, Mark P. Plummer, Clive N. May, Yugeesh R. Lankadeva

**Affiliations:** 1 Translational Cardiovascular and Renal Research Group and Preclinical Critical Care Unit, Florey Institute of Neuroscience and Mental Health, University of Melbourne, 30 Royal Parade, Parkville, 3052 Melbourne, VIC Australia; 2 Translational Neurodegeneration Laboratory, Florey Institute of Neuroscience and Mental Health, University of Melbourne, Melbourne, VIC Australia; 3 Department of Intensive Care, Royal Adelaide Hospital, Adelaide, Australia; 4 Department of Critical Care, Melbourne Medical School, University of Melbourne, Melbourne, VIC Australia

**Keywords:** Sepsis, Sodium ascorbate, Megadose, Acute kidney injury, Hypoxia, Ischemia, Vasopressor

## Abstract

**Background:**

Megadose sodium ascorbate has shown promise as a treatment to reverse the pathophysiological effects of ovine Gram-negative sepsis. In human septic shock, lower doses of sodium ascorbate improved urine output and reduced vasopressor requirements compared with placebo. We sought to determine the minimum therapeutic dose of sodium ascorbate required to reverse sepsis-induced cardiovascular and renal dysfunction in sheep.

**Methods:**

Healthy young adult sheep were instrumented with renal artery flow probes, and oxygen-sensing and laser Doppler probes in the kidneys. Non-anaesthetised animals were infused with live *Escherichia coli* for 31-h. At 23.5-h of sepsis, four groups (n = 7–8/group) received fluid resuscitation (30 mL/kg Hartmann’s solution) and were randomized to intravenous sodium ascorbate (1.0, 2.0, or 3.0 g/kg) or vehicle, delivered as a bolus followed by 7-h infusion. Norepinephrine was titrated to maintain mean arterial pressure (MAP) at ~ 70 mmHg.

**Results:**

At 23-h of sepsis, animals developed hypotension, hyperlactatemia, acute kidney injury, and renal medullary hypoxia. Vehicle-treated sheep required escalating doses of norepinephrine (from 0.4 to 0.8 ± 0.2 µg/kg/min) to restore MAP. Sodium ascorbate at 3.0 g/kg (achieving plasma ascorbate levels of ~ 10 mmol/L) rapidly restored MAP, allowing withdrawal of norepinephrine in half the animals (P = 0.007). Lower doses of sodium ascorbate (1.0 and 2.0 g/kg) had no significant effect on vasopressor requirements. The improvements in renal medullary oxygenation (25.2 ± 3.3 to 43.4 ± 4.5 mmHg, P = 0.04) and urine flow (from 0.5 ± 0.2 to 6.9 ± 2.4 ml/kg/h, P < 0.0001) were dose-dependent. Renal medullary tissue protein expression of nuclear factor kappa-light chain-enhancer B was significantly reduced with 3.0 g/kg of sodium ascorbate (to -52.9 ± 13.3%, P = 0.0005) and phosphorylated endothelial nitric oxide synthase at Ser-1177 was upregulated (to +219.5 ± 51.4%, P = 0.04) compared with vehicle-treated sheep.

**Conclusions:**

In established ovine Gram-negative sepsis, only 3.0 g/kg sodium ascorbate effectively restored cardiovascular and renal dysfunction, which was associated with suppression of renal inflammatory signalling and restoration of endothelial nitric oxide activity. These findings demonstrate a clear dose-dependent therapeutic threshold, where achieving plasma ascorbate concentrations of ~ 10 mmol/L is essential to elicit multi-organ protection.

## Introduction

Sepsis remains the leading cause of death in intensive care units with a mortality rate ranging from 15–30% in high-income countries to 50% or more in low-income countries [1]. It is estimated that there are 49 million cases and 11 million sepsis associated deaths each year worldwide [1]. Current treatments are largely supportive; there are no treatments that reverse sepsis-induced multiple organ dysfunction.

Plasma levels of ascorbate are often reduced in sepsis, and these low levels correlate with severity of illness [2–4]. Accordingly, treatment with intravenous ascorbic acid (a form of vitamin C) has been proposed as a treatment for sepsis and septic shock. A further rationale for the use of ascorbic acid is its potentially beneficial effects, as an antioxidant, anti-inflammatory, anticoagulant, immune stimulant and cofactor for endogenous norepinephrine and vasopressin synthesis [5, 6].

Several clinical trials and meta-analyses have evaluated the effects of intravenous ascorbic acid in sepsis, typically administering doses between 6 and 24 g/day. The rationale for the low-to-moderate doses of ascorbic acid was based on a Phase I safety trial comparing the safety and tolerability of 50 and 200 mg/kg/day of ascorbic acid in patients with sepsis [7]. Reported outcomes from clinical trials using low-to-moderate doses of ascorbic acid in sepsis have been inconsistent, with many trials showing no significant benefit [8, 9], mild benefit [10–12] or detrimental outcomes [13, 14]. In view of these mixed findings, interest in ascorbic acid as a therapy for sepsis has waned.

In a large animal model of Gram-negative sepsis, we investigated the effects of sodium ascorbate, the sodium salt of vitamin C, administered at doses far exceeding those previously tested in critically ill patients. Instead of ascorbic acid, we chose to use sodium ascorbate as we proposed that it would avoid aggravating the incipient metabolic acidosis, a known adverse effect of intravenous administration of titratable acidic formulation in sepsis [15, 16]. This consideration is clinically relevant, as metabolic acidosis is common in septic shock and independently associated with increased mortality [17]. In preclinical safety and efficacy studies, we showed that intravenous megadose sodium ascorbate (3.75 g/kg over 7-h) rapidly reversed all the pathophysiological effects of Gram-negative sepsis in non-anesthetised sheep [18, 19]. In a pilot double-blind randomized controlled trial in patients with septic shock, intravenous sodium ascorbate (60 g over 6-h) significantly increased urine output and produced greater reductions in vasopressor requirements and sequential organ failure assessment (SOFA) scores compared with placebo [20].

To inform the design of future multicentre clinical trials, this study aimed to identify the minimum intravenous dose of sodium ascorbate required for optimal therapeutic benefit in sepsis. We hypothesised that achieving very high plasma ascorbate concentrations is essential to reproduce its full multi-organ protective effects, and that the threshold dose for organ protection may differ across physiological systems. Accordingly, we evaluated whether lower doses of sodium ascorbate (1.0, 2.0 or 3.0 g/kg) could replicate the benefits previously observed with megadose (3.75 g/kg) using an established ovine model of Gram-negative sepsis. The dosing regimen (bolus followed by infusion from 24–31 h of sepsis) was designed to match the pharmacokinetic profile of sodium ascorbate in our prior translational studies, ensuring experimental consistency and translatability [18, 19]. The primary outcome was a reduction in norepinephrine requirements to attain target mean arterial pressure (MAP). Secondary outcomes included renal medullary tissue oxygenation, urine output and renal medullary tissue protein expression levels of nuclear factor kappa-light-chain-enhancer B (NF-κB) and phosphorylated endothelial nitric oxide synthase at Ser-1177 residue to assess inflammation and nitric oxide bioavailability.

## Methods

### Animals

Healthy young, untreated adult Merino ewes (1.5–2.0 years, 41.6 ± 0.7 kg, n = 30) were acclimatised to the laboratory environment (12 h light: 12 h dark cycles) in pens for at least one week prior to being moved to individual metabolic cages for experimentation. Sheep were allowed free access to water and fed 800 g of oaten chaff daily. All experimental procedures were conducted at The Florey Institute of Neuroscience and Mental Health (Melbourne, Australia) and were approved by the Animal Ethics Committee of the Florey Institute of Neuroscience and Mental Health (AEC 20–041) under the guidelines of the National Health and Medical Research Council and conformed with the ARRIVE 2.0 guidelines [21].

### Surgical preparation

All sheep underwent two aseptic surgeries 3–4 weeks apart under general anaesthesia. In the first surgery, carotid arterial loops were created for arterial blood sampling and measurement of arterial pressure, and a flow probe was implanted around the pulmonary artery to facilitate measurement of cardiac output. In the second surgery, sheep were surgically instrumented to enable the continuous recording of renal blood flow (RBF), tissue oxygen tension (pO_2_) and perfusion of the renal cortex and medulla and the frontal cortex of the brain, brain temperature and core body temperature, as previously reported [19, 22, 23]. Prior to the first incision and at approximately 24 and 48-h after each surgery, intramuscular injections of antibiotic (900 mg of procaine penicillin, Ilium Propercilin, Troy Laboratories, NSW, Australia) and analgesia (1 mg/kg, Ilium Flunixil, Troy Laboratories) were given. An additional dose of flunixin was given at 4–6-h after commencement of surgery. Antibiotic and analgesia were not given during the experimental periods.

### Experimental protocol for induction of sepsis

Following a 3–5 day recovery period from the second surgery, non-anaesthetised sheep underwent a 24-h baseline recording period. Gram-negative sepsis was then induced in non-anaesthetised sheep by intravenous infusion of a loading dose of live *E. coli* (2.8 × 10^9^ colony forming units (CFU)) over a 30-min period followed by a continuous infusion of 1.26 × 10^9^ CFU/h for the next 30.5-h. All animals received fluid bolus therapy with Hartmann’s solution (30 mL/kg over 30-min, Baxter, Australia) from 23 to 23.5-h after commencement of sepsis induction. In addition, norepinephrine was infused intravenously from 25 to 31-h of sepsis, at a dose titrated to maintain a target MAP of ~ 70 mmHg. For the duration of the experiment, analogue signals of cardiovascular, renal and cerebral variables were continuously acquired at 100 Hz using a CED Micro-1401 interface and Spike2 software (Cambridge Electronic Design, Cambridge, UK) [23].

### Intervention with sodium ascorbate

Animals were block-randomised to receive one of four treatments from 23.5 to 31-h of sepsis: 1) 3.0 g/kg sodium ascorbate (n = 8, body weight = 45.0 ± 1.0 kg), 2) 2.0 g/kg sodium ascorbate (n = 7, body weight = 40.6 ± 1.3 kg), 3) 1.0 g/kg sodium ascorbate (n = 7, body weight = 39.9 ± 1.4 kg) or 4) vehicle (fluid-matched to infusion of 3.0 g/kg of sodium ascorbate) (n = 8, body weight = 41.6 ± 3.8 kg). Sodium ascorbate (30 g/100 mL, Biological Therapies, Victoria, Australia) and vehicle were infused intravenously as a 1:1 dilution of 5% glucose in water (Baxter, Victoria, Australia).

At 23.5-h of sepsis, after fluid resuscitation (30 mL/kg Hartmann’s solution), sodium ascorbate was administered as a loading dose (0.5 g/kg sodium ascorbate over 30-min) followed by a continuous infusion of the remaining dose of sodium ascorbate from 24-h to 31-h of sepsis (*i.e.* 0.5, 1.5 or 2.5 g/kg in the 1.0, 2.0 and 3.0 g/kg groups, respectively). The vehicle group received a bolus dose of 2.5% glucose fluid-matched to the 0.5 g/kg sodium ascorbate bolus, followed by a continuous infusion of 2.5% glucose fluid-matched to the 2.5 g/kg sodium ascorbate infusion for the rest of the intervention duration. At the end of the treatment, animals were euthanised with intravenous sodium pentobarbitone (100 mg/kg; Lethaton, Randlab, NSW, Australia).

### Blood sampling and periodic measurements

Arterial blood, renal venous blood and urine samples were collected at baseline and at 23, 25, 27, 29 and 31-h after starting the infusion of *E. coli*. Blood gas oximetry and lactate concentrations in arterial and renal venous blood were determined at the same time-points (ABL System 625, Radiometer Medical, Denmark). Plasma and urinary concentrations of creatinine and sodium were determined at the Pathology Service at Austin Hospital, Victoria, Australia.

### Measurements of plasma ascorbate levels

Plasma samples (300 µL) were added to a 1.2 mL mixture of 90% v/v methanol and 313 µM of diethylenetriaminepentaacetic acid [20]. This induced deproteination of the plasma and chelated metals that may interfere with the measurement of ascorbate levels. The concentration of plasma ascorbate was determined fluorometrically using a microplate assay [19, 20].

### Western blot analysis for renal medullary tissue levels of NF-κB and eNOS

The renal medulla of sheep treated with 3.0 g/kg sodium ascorbate or its vehicle was isolated at necropsy and flash frozen in liquid nitrogen. The tissue was later mechanically homogenized and the protein concentrations of NF-κB, total eNOS and phosphorylated eNOS at Ser-1177 and Thr-495 residues were determined with western blot, as previously described [24].

### Statistical analysis

Data are expressed as mean ± standard error of mean. A Student’s paired t-test between the levels at baseline and 23-h of sepsis was conducted to determine the significant changes induced by sepsis. Data from continuous recordings of variables from 23 to 31-h of sepsis, were analysed with two-way repeated measures analysis of variance. P-values derived from within-subject factors were conservatively adjusted using the Greenhouse–Geisser method [25]. If P_Time_ and/or P_Treatment× Time_ was ≤ 0.05, Dunnett’s post-hoc test was used for within-group multiple comparisons. If P_Treatment_ and/or P_Treatment*Time_ was ≤ 0.05, then a Tukey’s post-hoc test was used for between group multiple comparisons of 1.0, 2.0, 3.0 g/kg sodium ascorbate and vehicle treatment groups at each time-point. To determine the effectiveness of the treatment, data from variables at 31-h sepsis were compared with baseline levels using a Student’s paired t-test. Statistical analyses and figures were generated using GraphPad Prism software (Version 10, La Jolla, USA). Two-sided P-values < 0.05 were considered statistically significant. We previously showed that there was an up-regulation in inflammation and eNOS was uncoupled in the renal tissue of septic sheep with AKI [24]. Thus, we conducted a one-tailed unpaired Student’s t-test to determine the specific hypotheses that sodium ascorbate treatment reduces the tissue protein expression of NF-κB and improves coupling state of eNOS and reduces uncoupling state of eNOS in the renal medulla of sepsis-induced AKI.

## Results

### Cardiovascular, renal and cerebral responses to sepsis in the vehicle group

**Fig. 1:**
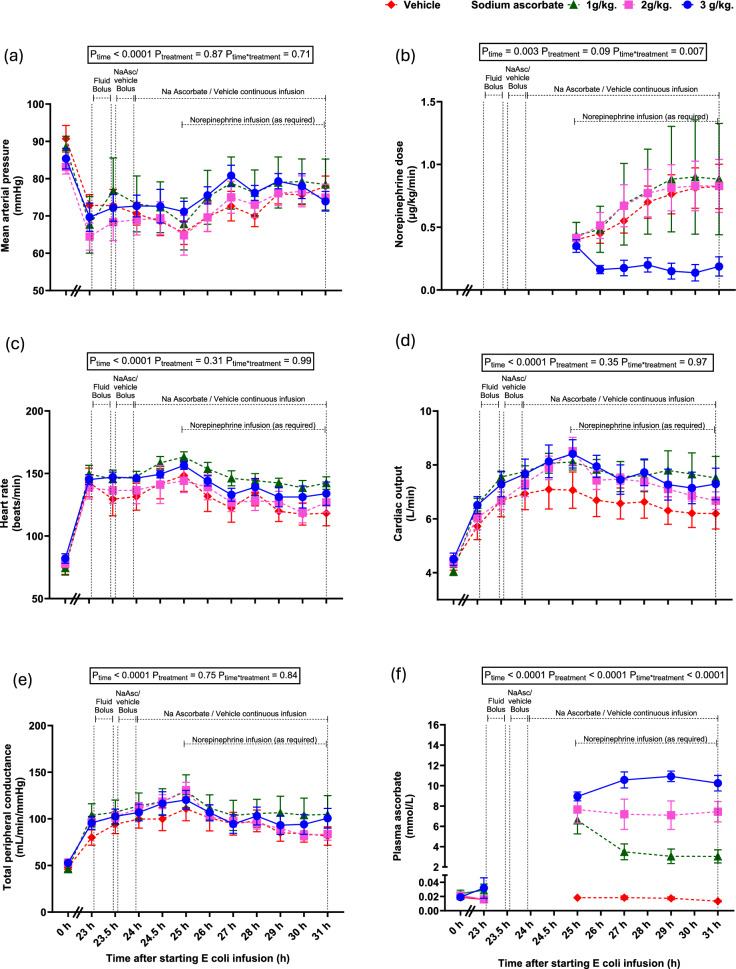
Cardiovascular effects of megadose sodium ascorbate and vehicle in ovine sepsis. Mean arterial pressure (**a**), heart rate (**c**), cardiac output (**d**) and total peripheral resistance (**e**) over a 24-h baseline period (0-h) and during infusion of live *E. coli* from 0 to 31-h. All animals received a fluid bolus (30 ml/kg Hartmann’s solution) from 23 to 23.5-h of sepsis. Animals were randomised to receive either sodium ascorbate (3.0 g/kg (n = 8); 2.0 g/kg (n = 7); 1.0 g/kg (n = 7)) or vehicle (n = 8) from 23.5 to 31-h of sepsis. Sodium ascorbate was infused as a bolus (0.5 g/kg over 30 min) with the remainder of each dose given from 24 to 31-h of sepsis. Norepinephrine (**b**) was given as required from 25 to 31-h of sepsis to achieve a target mean arterial pressure of 70–80 mmHg. Plasma ascorbate concentrations were determined before and at pre-determined time-points during the 7-h intervention period (**f**). Data are presented as mean ± standard error of mean. P-values are outcomes of a 2-way repeated measures of analysis of variance from 23 to 31-h of sepsis

By 23-h of intravenous infusion of *E. coli*, hyperdynamic sepsis developed, characterised by hypotension, peripheral vasodilatation, tachycardia and increased cardiac output (all P < 0.0001, Table 1, Fig. 1). Progressively increasing doses of norepinephrine were required from 25–31-h of sepsis in the vehicle group (from 0.4 to 0.8 ± 0.2 µg/kg/min, P_time_ = 0.003) to maintain MAP at ~ 70 mmHg (Fig. 1b).

**Table 1:** Systemic, renal and cerebral variables at baseline and at 23-h after induction of sepsis

Variables	Pooled (n = 30)		3.0 g/kg Na ascorbate (n = 8)
	Baseline	23-h of sepsis (prior to treatment)	31-h of sepsis (end of treatment)
*Systemic *			
Mean arterial pressure (mmHg)	87.0 ± 8.1	68.8 ± 2.3 ****	74.0 ± 2.6*
Heart rate (beats/min)	77.4 ± 2.2	144.1 ± 4.0 ****	133.9 ± 9.3**
Cardiac output (L/min)	4.3 ± 0.6	6.2 ± 0.2 ****	7.3 ± 0.6**
Total peripheral resistance (ml/min/mmHg)	49.7 ± 1.2	93.3 ± 4.5****	100.6 ± 10.5**
Core body temperature (℃)	39.7 ± 0.1	41.5 ± 0.1 ****	39.7 ± 0.2
Arterial blood lactate (mmol/L)	0.63 ± 0.04	1.50 ± 0.1 ****	1.5 ± 0.3
Arterial blood pH	7.52 ± 0.01	7.54 ± 0.005****	7.49 ± 0.01
*Renal*			
Renal blood flow (mL/min)	248.9 ± 9.6	330.0 ± 16.9 ****	387.3 ± 34.3**
Medullary tissue pO_2_ (mmHg)	43.6 ± 1.6	24.3 ± 2.0 ****	43.4 ± 4.5
Medullary tissue perfusion (BPU)	1143 ± 140	650 ± 70 ***	892.3 ± 203.5
Cortical tissue pO_2_ (mmHg)	38.8 ± 2.3	43.2 ± 2.9	40.5 ± 6.4
Cortical tissue perfusion (BPU)	2140 ± 343	1931 ± 314	2547 ± 213
Urine output (mL/kg/h)	1.47 ± 0.13	0.52 ± 0.10 ****	6.92 ± 0.84***
Plasma creatinine (µmol/L)	67.1 ± 2.0	141.8 ± 8.9 ****	18.8 ± 3.8****
Creatinine clearance (mmol/kg/min)	2.15 ± 0.13	0.86 ± 0.10 ****	36.6 ± 13.0*
*Cerebral*			
Cerebral tissue pO_2_ (mmHg)	35.7 ± 1.5	17.3 ± 2.1 ****	31.2 ± 1.6
Cerebral tissue perfusion (BPU)	978 ± 164	1045 ± 173	652.7 ± 150.4
Cerebral tissue temperature (℃)	39.5 ± 0.1	40.8 ± 0.1 ****	39.5 ± 0.3

BPU: blood perfusion units. Data are expressed as mean ± standard error of mean. P-values were derived from a Student’s paired t-test comparing either 23.5-h or 31-h of sepsis to their respective baseline levels. *P ≤ 0.05, **P < 0.01, *** P < 0.001, **** P < 0.0001

**Fig. 2:**
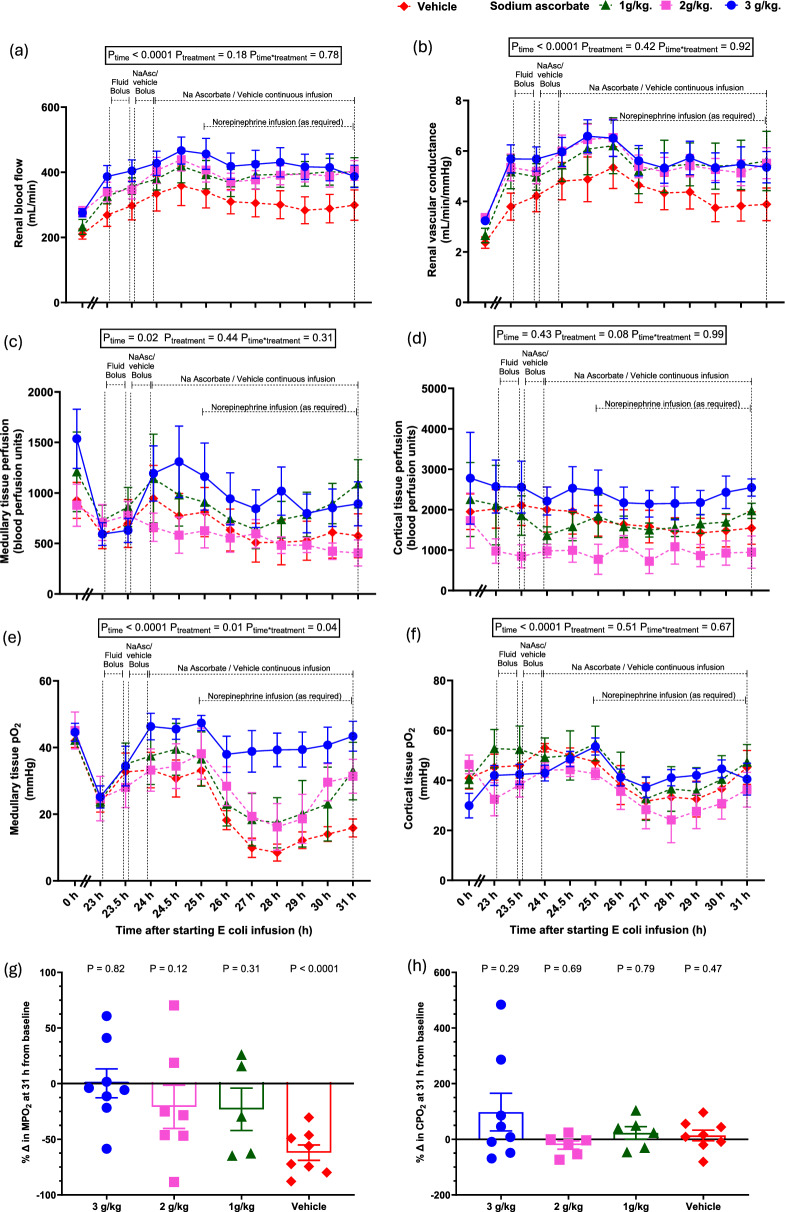
Renal effects of megadose sodium ascorbate in ovine sepsis. Renal blood flow (**a**), renal vascular conductance (**b**), medullary tissue perfusion (**c**) and oxygen tension (**e**), and renal cortical tissue perfusion (**d**) and oxygen tension (**f**) during infusion of live *E. coli* from 0 to 31-h. The percentage changes in medullary and cortical tissue oxygen tension at 31-h from their respective baseline levels are represented in (**g-h**). Data are presented as mean ± standard error of mean. P-values are outcomes of a 2-way repeated measures of analysis of variance from 23 to 31-h of sepsis in (**a-f**) and Student’s paired t-test from 31-h sepsis to their baseline levels in (**g-h**). Fluid and drug infusion regimens and group numbers are as detailed in Fig. 1

**Fig. 3:**
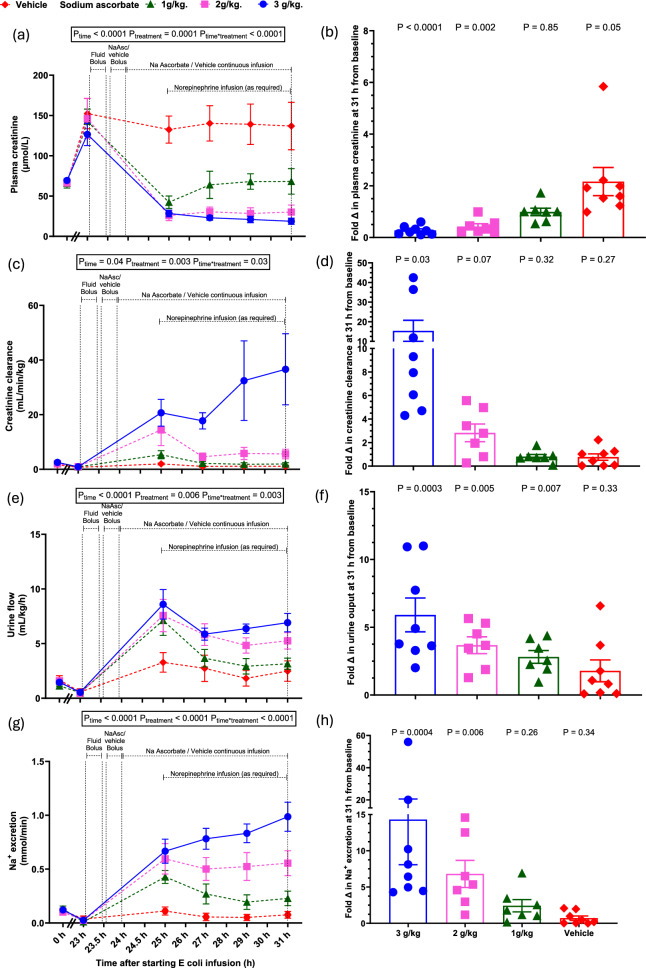
The effects of megadose sodium ascorbate on renal function in ovine sepsis. Plasma creatinine (**a**), creatinine clearance (**c**), urine output (**e**) and urinary excretion of sodium (**g**) during infusion of live *E. coli* from 0 to 31-h. Fold changes from their respective baselines at 31-h of sepsis in plasma creatinine (**b**), creatinine clearance (**d**), urine output (**f**) and sodium excretion (**h**). Data are presented as mean ± standard error of mean. P-values are outcomes of a 2-way repeated measures of analysis of variance from 23 to 31-h of sepsis in (**a, c, e, g**) and Student’s paired t-test from 31-h sepsis to their baseline levels in (**b, d, f, h**). Fluid and drug infusion regimens and group numbers are as detailed in Fig. 1

In the vehicle group, at 23-h of sepsis, there were significant reductions in renal medullary tissue pO_2_ and perfusion, despite sustained increases in renal vascular conductance and RBF, with these changes being maintained until 31-h of sepsis (Table 1, Fig. 2a, b, c, e). In contrast, renal cortical tissue pO_2_ and perfusion were well-maintained (Table 1, Fig. 2d, f). Over this time there was a sustained increase in plasma creatinine and decrease in creatinine clearance and in urine flow (all P < 0.0001, Table 1, Fig. 3), indicative of development of Stage 2 acute kidney injury (AKI) according to the Kidney Disease: Improving Global Outcomes (KDIGO) criteria [26]. Sepsis-induced microcirculatory and AKI persisted from 24 to 31-h of sepsis.

Throughout the period of established sepsis in the vehicle group, cerebral tissue pO_2_ was reduced, and cerebral temperature increased (both P < 0.0001, Table 1, Fig. 6a, e). These changes were accompanied by typical sickness behaviour, including malaise, lethargy and unresponsiveness to external stimuli.

By 23-h of sepsis, arterial blood lactate had significantly increased in the vehicle group with a further increase by the end of the experiment (P < 0.0001, Table 1, Fig. 7a). Sheep were febrile with the increase in body temperature being maintained throughout the period of established sepsis (P < 0.001, Table 1, Fig. 6g).

### Plasma ascorbate concentrations with megadose sodium ascorbate

Infusion of sodium ascorbate produced marked, dose-dependent increases in plasma ascorbate levels (Fig. 1f). After the bolus dose and 1-h of infusion, plasma concentrations were 8.9 ± 0.4, 7.7 ± 1.3, and 6.6 ± 1.3 mmol/L in the 3.0, 2.0, and 1.0 g/kg groups, respectively. These levels were sustained to 31-h in the 3.0 and 2.0 g/kg groups (10.3 ± 0.7 and 7.45 ± 1.00 mmol/L) but declined in the 1.0 g/kg group (2.9 ± 0.7 mmol/L). Plasma ascorbate remained unchanged at lower levels in vehicle-treated animals.

### Norepinephrine requirements with megadose sodium ascorbate: Primary outcome

By 23-h of sepsis, there was a similar decrease in MAP in all groups (Fig. 1a). Vehicle-treated sheep and those receiving 1.0 or 2.0 g/kg sodium ascorbate required progressively escalating doses of norepinephrine (0.83 ± 0.3, 0.88 ± 0.4 and 0.83 ± 0.2 µg/kg/min respectively) over the 7-h intervention period to maintain MAP (Fig. 1a–b, P_time*treatment_ = 0.007). In contrast, 3.0 g/kg sodium ascorbate rapidly reduced norepinephrine requirements to 0.2 ± 0.1 µg/kg/min at 31-h (P_time*treatment_ = 0.007), with complete weaning off norepinephrine in 4 of 8 animals (Fig. 1b).

### Renal medullary tissue oxygenation and kidney function with megadose sodium ascorbate: secondary outcomes

The highest dose (3.0 g/kg) rapidly reversed renal medullary hypoxia to baseline values, with this effect being sustained throughout the experiment (from 25.2 ± 0.002 at 23-h to 43.4 ± 0.8 mmHg at 31-h, P = 0.006, Fig. 2e, g). The two lower doses induced partial improvements in medullary tissue pO₂, which waned during infusion but recovered by 31-h to pre-treatment sepsis levels (Fig. 2e, g). None of the doses altered renal cortical perfusion or oxygenation (Fig. 2d, f, h). None of the doses of sodium ascorbate altered sepsis-induced renal vasodilatation or the increase in renal blood flow (Fig. 2a-b). Sodium ascorbate caused a large dose-related increase in urine flow (from 0.5 ± 0.2 at 23-h to 6.9 ± 0.8 ml/kg/h at 31-h in 3.0 g/kg, P_time*treatment_ < 0.0001, Fig. 3e, Table 2). In contrast, all three doses significantly attenuated the rise in plasma creatinine, with levels returning to or below baseline from 1.5 h of treatment (P_time*treatment_ < 0.0001, Fig. 3a–b). Creatinine clearance increased in a dose-dependent manner, with the greatest effect at 3.0 g/kg (P_time*treatment_ = 0.03, Fig. 3c–d, Table 2).

**Table 2:** Summary of the effectiveness of 7-h of sodium ascorbate treatment to normalise sepsisinduced changes in renal, cerebral and systemic variables to their pre-morbid levels

Variables	Dose of ascorbate			
	3.0 g/kg	2.0 g/kg	1.0 g/kg	Vehicle
*Renal*				
Plasma creatinine (µmol/L)	+ + +	+ +	+	-
Creatinine clearance (mmol/min)	+ + +	+ +	-	-
Medullary tissue pO_2_ (mmHg)	+ + +	+ +	+	-
Medullary tissue perfusion (BPU)	-	-	-	-
Urine flow (mL/kg/h)	+ + +	+ +	+	-
*Cerebral*				
Cerebral tissue pO_2_ (mmHg)	+	-	-	-
Cerebral tissue temperature (℃)	+	+	+	-
*Systemic*				
Noradrenaline required	+	-	-	-
Heart rate (beats/min)	-	-	-	-
Cardiac output (L/min)	-	-	-	-
Core body temperature (℃)	+	+	+	-
Arterial blood lactate (mmol/L)	+	+	+	-
Arterial blood pH	-	-	-	-

‘ + ’ indicates normalisation of variable at 31-h to its pre-morbid levels and the number of ‘ + ’ indicates magnitude of improvements. ‘-’ indicates no significant improvement detected

### Renal medullary tissue protein levels of NF-κB and eNOS with megadose sodium ascorbate: secondary outcomes

**Fig. 4:**
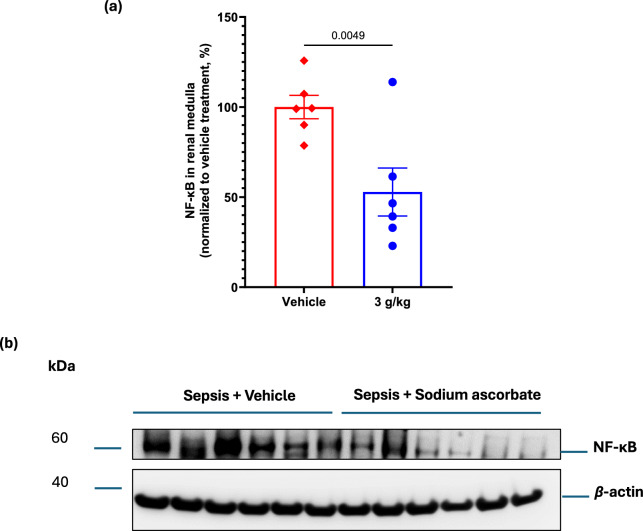
Protein expression of nuclear factor kappa-light-chain-enhancer of activated B cells (NF-κB) in renal medulla tissue of septic sheep treated with 3.0 g/kg sodium ascorbate (blue bars, n = 6) or its vehicle (red bars, n = 6) (**a**). Data are presented as mean ± standard error of mean relative to b-actin and normalized to vehicle treatment (**b**). P-value is the outcome of 1-tailed unpaired t-test testing for the specific hypothesis that sodium ascorbate treatment reduces sepsis-induced upregulation of NF-κB

**Fig. 5:**
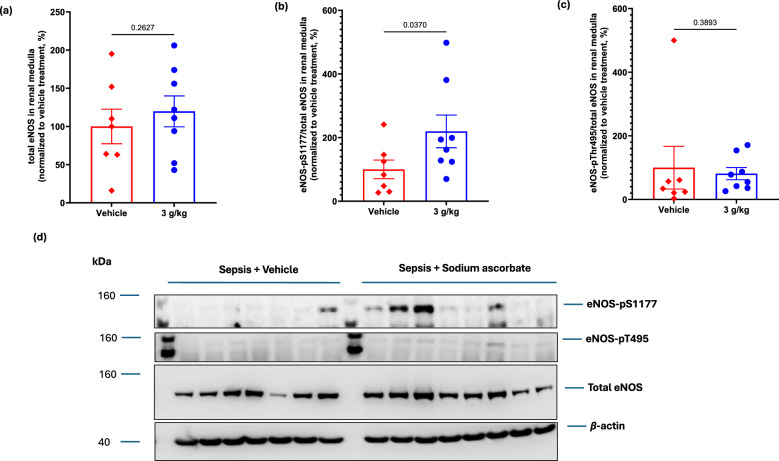
Protein expression of total endothelial nitric oxide synthase (eNOS, **a**) and its phosphorylation at Ser-1177 (**b**) and Thr-495 (**c**) residues in renal medulla tissue of septic sheep treated with 3.0 g/kg sodium ascorbate (blue bars, n = 6) or its vehicle (red bars, n = 6). Data are presented as mean ± standard error of mean relative to b-actin and normalised to vehicle treatment (**d**). P-values are outcomes of 1-tailed unpaired t-test testing for the specific hypothesis that sodium ascorbate treatment improves the state of coupling of eNOS

Renal medullary tissue protein expression of NF-κB, a protein complex that acts as a master regulator of transcription of many genes involved in inflammation, was significantly reduced with 3.0 g/kg of sodium ascorbate to −52.9 ± 13.3% (P = 0.005) compared with the levels in vehicle-treated animals (Fig. 4a, b). Renal medullary protein expression of eNOS phosphorylated at Ser-1177, a post-translational modification that increases eNOS enzyme activity and nitric oxide bioavailability, was significantly upregulated by sodium ascorbate (3.0 g/kg) to 219.5 ± 51.4% of the level with vehicle treatment (P = 0.04, Fig. 5b, d). In contrast, renal medullary tissue levels of total eNOS and eNOS phosphorylated at Thr-495 (P = 0.39) were not significantly altered by sodium ascorbate (3.0 g/kg) (Fig. 5a, c, d).

### Cerebral oxygenation and sickness behaviour with megadose sodium ascorbate

**Fig. 6:**
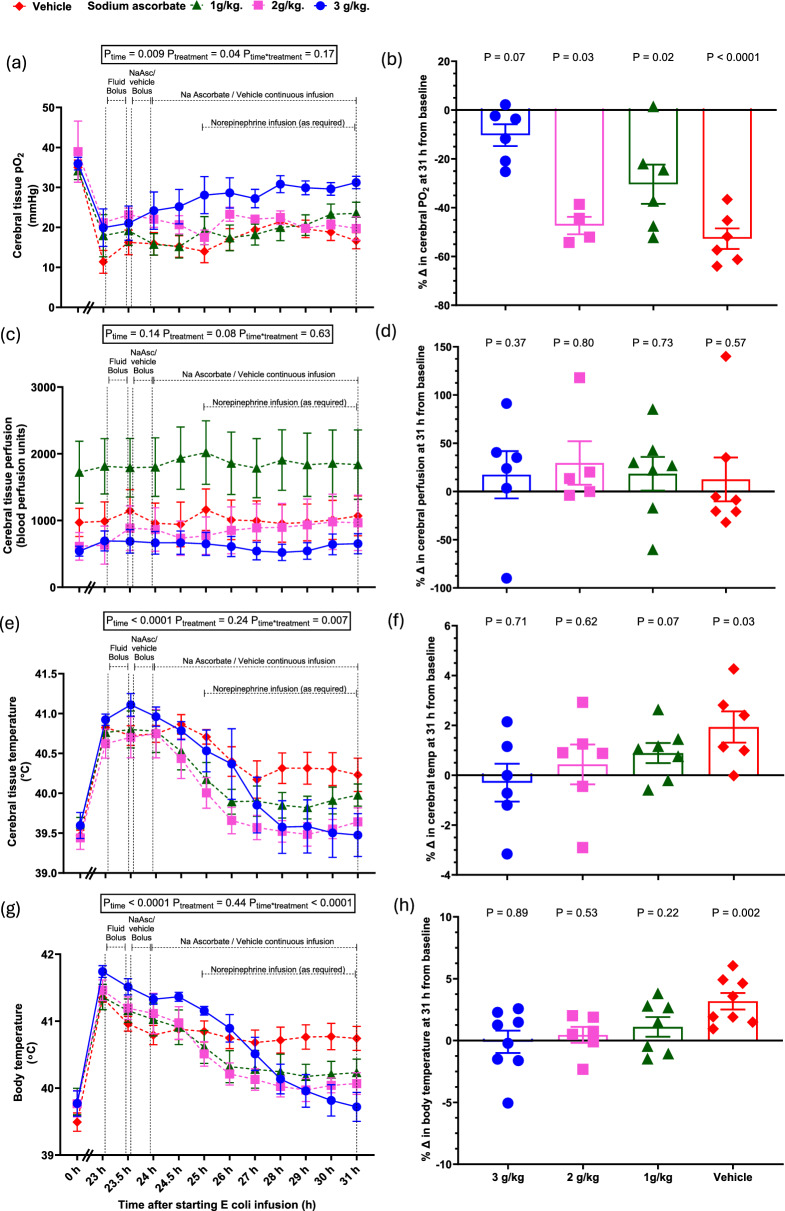
Cerebral effects of megadose sodium ascorbate in ovine sepsis. Cerebral tissue pO_2_ (**a**), perfusion (**c**) and temperature (**e**), and core body temperature (**g**) during infusion of live *E. coli* from 0 to 31-h. Percentage changes at 31-h of sepsis from their respective baselines in cerebral tissue pO_2_ (**b**), perfusion (**d**) and temperature (**g**), and core body temperature (**h**). Data are presented as mean ± standard error of mean. P-values are outcomes of a 2-way repeated measures of analysis of variance from 23 to 31-h of sepsis in (**a, c, e, g**) and Student’s paired t-test from 31-h sepsis to their baseline levels in (**b, d, f, h**). Fluid and drug infusion regimens and group numbers are as detailed in Fig. 1

In vehicle-treated animals, cerebral hypoxia, hyperthermia, and sickness behaviours persisted (Fig. 6a–f). In contrast, 3.0 g/kg sodium ascorbate restored normal behaviour within 3 to 4-h, with animals standing, responding to stimuli, and resuming feeding and drinking. This clinical recovery paralleled normalization of cerebral tissue pO₂ (from 19.9 ± 4.7 mmHg at 23-h to 31.2 ± 1.6 mmHg at 31-h; baseline 35.9 ± 1.6 mmHg, P = 0.10; Fig. 6a–b). No improvements in behaviour or cerebral oxygenation were observed with the lower doses of sodium ascorbate (1.0 or 2.0 g/kg, Table 2). Cerebral perfusion was unchanged by sepsis and unaffected by any of the doses of sodium ascorbate (Fig. 6c–d). All doses normalized cerebral and core body temperature (Fig. 6e–g, Table 2).

### Arterial blood gases and biochemistry with megadose sodium ascorbate

**Fig. 7:**
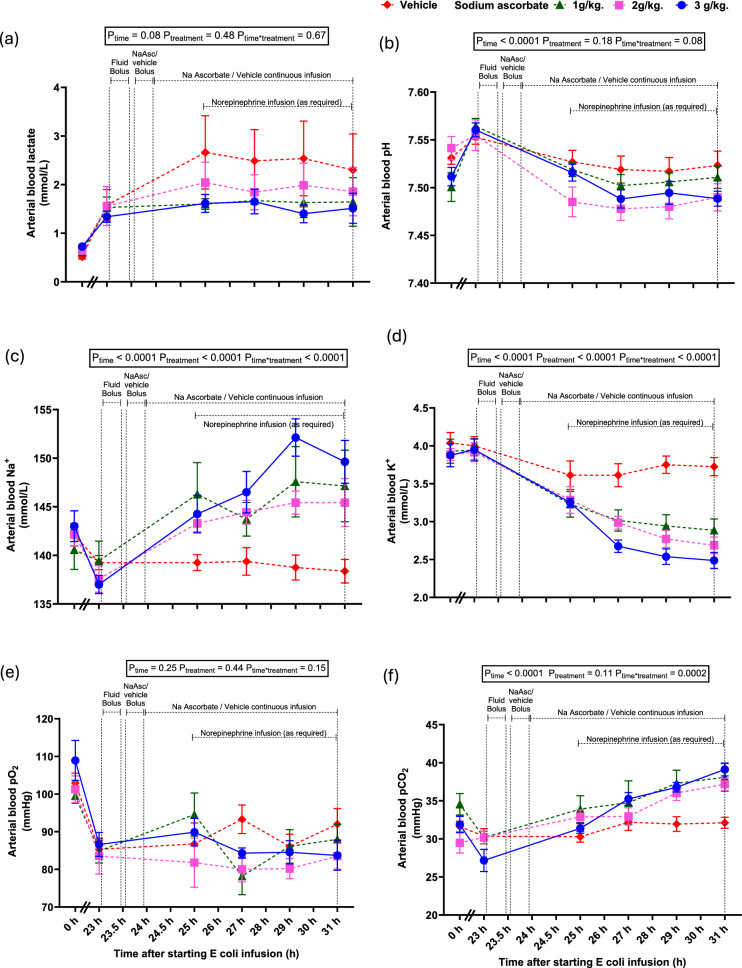
The effects of megadose sodium ascorbate on arterial blood chemistry in ovine sepsis. Arterial blood concentrations of lactate (**a**), sodium (**c**) potassium (**d**) pH (**d**), pO_2_ (**e**) and pCO_2_ (**f**) during infusion of live *E. coli* from 0 to 31-h. Data are presented as mean ± standard error of mean. P-values are outcomes of a 2-way repeated measures of analysis of variance from 23 to 31-h of sepsis. Fluid and drug infusion regimens and group numbers are as detailed in Fig. 1

At 23 h of sepsis, blood lactate was increased ~ 2.5-fold in all groups (Table 1, Fig. 7a). Lactate continued to rise in vehicle-treated animals but was stabilized by all doses of sodium ascorbate, though not restored to baseline (Fig. 7a). Arterial pH and bicarbonate remained unchanged with vehicle. In the 3.0 g/kg group, pH was unchanged (7.51 ± 0.01 to 7.49 ± 0.01, P = 0.06), but bicarbonate rose significantly (25.2 ± 0.67 to 29.43 ± 0.77 mmol/L, P = 0.006). Sodium ascorbate increased arterial sodium and reduced potassium (Fig. 7c–d). Arterial pO₂ was unaffected, while pCO₂ increased modestly (Fig. 7e–f).

## Discussion

The main finding of this study is that in ovine Gram-negative sepsis, only the highest megadose of intravenous sodium ascorbate (3.0 g/kg) effectively enabled withdrawal of vasopressor support, improved renal function, and restored animals to a normal, healthy clinical state. This dose achieved a plasma ascorbate concentration of ~ 10 mmol/L, which is substantially higher than the levels reported in preclinical and clinical studies of vitamin C in sepsis. In contrast, the lowest dose tested (1.0 g/kg), which still exceeded the doses administered in prior trials, conferred only modest benefit. These results strongly suggest that the inconsistent or absent therapeutic effects of vitamin C used as ascorbic acid in sepsis, reported in earlier clinical studies, may be attributable to the use of doses that were insufficient to produce the high plasma concentrations required for multiple organ protection.

### Dose related effects of sodium ascorbate

The reversal of the sepsis-induced pathophysiological changes achieved with the highest dose of sodium ascorbate (3.0 g/kg) in this study is consistent with our previous preclinical work using a slightly higher dose (3.75 g/kg) [18, 19]. Importantly, these effects cannot be attributed to the sodium load, as they were not replicated by an equimolar intravenous infusion of sodium bicarbonate [19]. Taken together, these findings suggest that 3.0 g/kg sodium ascorbate lies near the upper plateau of the dose–response curve. Administered as a bolus followed by a continuous infusion, this regimen maintained stable plasma ascorbate concentrations of ~ 10 mmol/L. These results indicate that clinical trials of sodium ascorbate in patients with sepsis should be designed to achieve similarly high plasma ascorbate levels to maximize therapeutic benefits.

Interestingly, the threshold dose required to elicit the beneficial effects of sodium ascorbate varied across organ systems. The reduction in norepinephrine requirement, reversal of cerebral hypoxia, and restoration of normal clinical behaviour was only observed with the highest dose (3.0 g/kg). In contrast, even the lowest dose (1.0 g/kg) reduced body temperature, while improvements in renal medullary oxygenation, renal function, and arterial lactate were dose dependent. These findings suggest that different physiological systems require distinct tissue concentrations of ascorbate to counteract sepsis-induced multiple organ dysfunction, consistent with the involvement of organ-specific pathophysiological mechanisms.

### Relationship with previous studies

Comparison with other preclinical and clinical studies is challenging, as most have investigated ascorbic acid rather than sodium ascorbate, and at substantially lower doses. Apart from our recent clinical trial [20], sodium ascorbate has not been evaluated for sepsis or septic shock. In that pilot double-blind randomized controlled trial in patients with septic shock, a single 60 g intravenous infusion of sodium ascorbate administered over 6-h significantly increased urine output and reduced vasopressor requirements and SOFA score compared with placebo. The peak plasma concentration achieved was 5.7 mmol/L, similar to that observed with 1.0 g/kg in septic sheep, which produced only modest renal improvements and no effect on arterial lactate, closely mirroring the limited clinical responses [20].

In contrast, the majority of clinical studies have used intravenous doses of ascorbic acid between 6 and 24 g/day. Results have been inconsistent, with reports of both benefit and harm. Potential efficacy was suggested in an early placebo-controlled trial, ascorbic acid (50 or 200 mg/kg/day for 4 days) reduced SOFA scores and inflammatory markers in patients with severe sepsis [7]. Several subsequent trials using 25–200 mg/kg/day for 3–4 days also demonstrated beneficial effects [10–12]. However, two large, recent clinical trials using 50 mg/kg every 6-h for 96-h in septic and COVID-19 patients reported higher rates of death or persistent organ dysfunction at 28 days in the ascorbic acid groups compared with placebo [13, 14].

Plasma ascorbate was only measured in a few of these studies. In patients with septic shock, infusion of vitamin C at 200 mg/kg/h increased plasma ascorbate concentrations to 3.08 mmol/L, and after 1.5 g every 6-h, the median peak plasma concentration was 0.37 mmol/L [7, 27]. These plasma levels are significantly lower than those that were required in the present study (10 mmol/L) to show multiple organ benefits in sepsis.

### Tissue oxygenation

In addition to restoring vascular responsiveness to norepinephrine, a striking effect of megadose sodium ascorbate was the normalization of tissue oxygenation in both the renal medulla and cerebral cortex. Renal medullary hypoxia has been proposed as a key driver of septic AKI [24, 28], and our finding that renal function improved when medullary pO₂ was restored supports this concept. Notably, reversal of medullary hypoxia and renal dysfunction occurred only with 3.0 g/kg of sodium ascorbate, which also enabled norepinephrine withdrawal. In contrast, with the two lower doses escalating norepinephrine requirements were associated with declining medullary pO₂, consistent with our previous observation that norepinephrine exacerbates renal medullary tissue hypoxia in ovine septic AKI [29].

Similarly, only the highest dose of sodium ascorbate restored cerebral pO₂ and normalized brain temperature, and this was accompanied by a marked recovery in clinical behaviour—from lethargy and sickness behaviour to alertness, mobility, and feeding. Together, these findings demonstrate that sepsis-induced tissue hypoxia in the renal medulla and frontal cerebral cortex likely contributes to the development of AKI and deterioration in clinical state, and that correction of this tissue hypoxia by megadose sodium ascorbate is associated with meaningful functional recovery.

### Biochemical changes

A key distinction of the present study, beyond the substantially higher doses than previously tested in critically ill patients, is the use of sodium ascorbate rather than ascorbic acid. Sodium ascorbate is pH-neutral (pH of 6.5–7.2), whereas the preparation of ascorbic acid used in the LOVIT trial has an acidic pH of 5.4–5.6 [15]. At the highest effective dose of sodium ascorbate, we did not observe a fall in blood pH or base excess, which remained well within the physiological range. To our knowledge, no clinical studies have reported the impact of intravenous ascorbic acid on arterial blood pH or base excess, although metabolic acidosis has been observed with ascorbic acid administration in cats [30]. A key future direction will be a direct comparison between equimolar doses of sodium ascorbate (3.0 g/kg) and ascorbic acid in ovine sepsis. This follow-up study will systematically examine the potential differences in efficacy, safety, and underlying physiological and biochemical mechanisms between the two formulations.

Consistent with earlier preclinical and clinical trials [18–20], sodium ascorbate produced hypernatremia at all three doses tested. However, this effect was offset by a robust, dose-dependent natriuresis, such that the rise in plasma sodium concentration was similar across doses. In contrast to our prior studies of megadose sodium ascorbate in sepsis, we did not observe improvements in arterial pO₂. Whether this reflects the slightly lower maximal dose used here (3.0 vs. 3.75 g/kg) or inter-group variability remains uncertain.

### Effects of sodium ascorbate on inflammation and nitric oxide bioavailability

NF-κB is a key upstream mediator for synthesis of pro-inflammatory cytokines including IL-6. Our observation that the highest dose of sodium ascorbate (3.0 g/kg) reduced the renal medullary NF-κB levels may, in part, explain our previous finding that sodium ascorbate attenuated the sepsis-induced rise in plasma IL-6 [19]. This suggest that sodium ascorbate exerts anti-inflammatory effects at the tissue level, contributing to improved renal function in sepsis.

The bioavailability of nitric oxide, a potent vasodilator, is critically dependent on the coupling state of eNOS. When eNOS is uncoupled, this can lead to reduced signalling and bioavailability of nitric oxide, which can lead to microcirculatory dysfunction [31–33]. We have previously demonstrated that sepsis selectively upregulates uncoupled eNOS, phosphorylated at the Thr-495 phosphorylation site in the renal medulla [24], suggesting that sepsis-induced renal microcirculatory dysfunction and AKI is partly driven by impaired nitric oxide bioavailability, In the present study, sodium ascorbate markedly increased phosphorylation of eNOS at the Ser-1177 residue, the activation site associated with proper enzyme coupling, which can enhance nitric oxide signalling and bioavailability. This mechanism likely contributed to improved renal microcirculation, ameliorating renal medullary tissue hypoxia and supporting recovery of kidney function.

We chose not to measure markers of oxidative or nitrosative stress in the current study based on our previous findings in the same ovine model of sepsis [24]. In that work, we observed an absence of classical oxidative and nitrosative stress, with plasma and renal tissue markers such as malondialdehyde and 3-nitrotyrosine being downregulated. This was accompanied by upregulation of antioxidant defence pathways, including increased renal tissue nuclear factor erythroid 2-related factor 2 protein expression, compared with healthy controls [24]. These findings challenge the conventional view that sepsis universally induces oxidative stress and suggest that reductive stress may be a more relevant pathological feature in this model, a hypothesis that warrants future molecular investigations.

### Strengths and limitations

Strengths included the use of live *E. coli* isolated from a patient with Gram-negative sepsis, instead of a laboratory bacterial strain or lipopolysaccharide. Treatment was started at 24-h of sepsis, when sepsis-induced multiple organ dysfunction was established. Time zero, the initiation of the septic insult, is known, whereas clinically it is usually unknown. Studies were performed in non-anesthetised sheep to remove the confounding effects of anaesthesia, although unlike many septic patients, the animals were not sedated or mechanically ventilated. A limitation of this study is that the sheep did not have pre-existing comorbidities and did not receive co-medications commonly used in the management of sepsis, besides fluid resuscitation and noradrenaline therapy. As a result, the model does not capture important clinical variables such as age, chronic cardiovascular or renal disease, diabetes, immunosuppression, or exposure to antimicrobial, or sedative agents that may influence disease trajectory or therapeutic responsiveness.

## Conclusions

We demonstrate that a megadose of intravenous sodium ascorbate (3.0 g/kg) was essential to enable withdrawal of vasopressor support, reverse renal medullary and cerebral tissue hypoxia, restore kidney function, and normalize the clinical state in young, healthy sheep with established sepsis induced by infusion of live *E. coli*. Notably, this megadose of sodium ascorbate did not cause metabolic acidosis, though it did increase plasma sodium. These findings indicate that achieving plasma ascorbate concentrations of ~ 10 mmol/L is critical to reverse the pathophysiological effects of sepsis. We are currently recruiting into a multi-center Phase Ib trial using this same dosing regimen in critically ill patients with septic shock (ANZCTRN12625001401448).

## Data Availability

The datasets used and/or analysed for this study are available from the corresponding author on reasonable request.

## Abbreviations

AKI: Acute kidney injury
ARRIVE: Animal research: reporting of in vivo experiments
CFU: Colony forming units
KDIGO: Kidney disease: improving global outcomes
MAP: Mean arterial pressure
pO_2_: Partial pressure of oxygen
RBF: Renal blood flow
SOFA: Sequential organ failure assessment score
